# Predictors and Outcomes of Non-Small Cell Lung Carcinoma Patients Following Severe Immune Checkpoint Inhibitor Toxicity: A Real-World UK Multi-Centre Study

**DOI:** 10.3390/cancers17172819

**Published:** 2025-08-28

**Authors:** Umair Mahmood, Eleni Josephides, Meenali Chitnis, Michael Skwarski, Spyridon Gennatas, Sharmistha Ghosh, James Spicer, Eleni Karapanagiotou, Tanya Ahmad, Martin Forster, Mariam Jamal-Hanjani, Sarah Benafif, Charles Swanton, Siow-Ming Lee, Dionysis Papadatos-Pastos, Alexandros Georgiou, Nicholas Coupe

**Affiliations:** 1 Oxford University Hospitals NHS Foundation Trust, Oxford OX3 7LJ, UK; 2 Guy’s and St. Thomas’ NHS Foundation Trust, London SE1 3SS, UK; 3 Department of Medical Oncology, University College London Hospitals NHS Foundation Trust, London NW1 2PG, UK; 4 Guy’s Hospital, King’s College London, London SE1 1UL, UK; 5 Cancer Metastasis Laboratory, University College London Cancer Institute, London WC1E 6DD, UK; 6 Cancer Research UK Lung Cancer Centre of Excellence, University College London Cancer Institute, London WC1E 6DD, UK; 7 Cancer Evolution and Genome Instability Laboratory, The Francis Crick Institute, London NW1 1AT, UK

**Keywords:** non-small cell lung carcinoma, immunotherapy, toxicity, progression-free survival, overall survival

## Abstract

Purpose: Evaluation of predictors and outcomes in NSCLC patients treated with an immune checkpoint inhibitor (ICI) following a severe immune-related adverse event (irAE). Methods: We included all NSCLC patients receiving ≥1 ICI cycle and corticosteroids for CTCAE Grade ≥3 irAEs between 2017 and 2023 from three UK NHS teaching hospitals. Progression-free survival (PFS) and overall survival (OS) after the 1st irAE, best overall response (BOR) to ICI, and predictors of clinical benefit were evaluated. Kaplan–Meier, Cox and logistic regression models, and Wilcoxon tests were used. Results: We screened 1658 NSCLC patients and identified 80 eligible subjects. The majority of patients had metastatic (*n* = 50, 63%) vs. localized (*n* = 30, 37%) NSCLC. Most patients developed a single ≥Grade 3 irAE on 1st line ICI (*n* = 71, 89%). Overall, 14 (18%) patients developed 2nd irAEs, 7 after rechallenge with ICIs. In the complete cohort, median OS after 1st irAE was 15.84 months (95% CI, 12.45–26.91). Lower neutrophil-to-lymphocyte ratio (NLR), patients receiving >4 cycles of ICI or median duration of ICI of >2.76 months before 1st irAE were associated with improved OS (*p* < 0.05), the latter two with PFS (*p* < 0.05). Age, gender, stage, *KRAS* mutation, PD-L1 and ICI type were not associated with PFS or OS. Pneumonitis as 1st irAE had the worst PFS and OS (*p* < 0.05). Median starting corticosteroid dose of ≤60 mg for 1st irAE had an improved OS (*p* = 0.04). Post 1st irAE response associated with better PFS and OS (*p* < 0.05). Number and duration of irAEs and additional immunosuppressive agents (14% of patients) were not associated with PFS or OS. Conclusions: In ≥Grade 3 irAEs patients managed with corticosteroids, lower baseline NLR, longer ICI use, response to ICI after 1st irAE, and a ≤60 mg corticosteroid dose had promising outcomes.

## Simple Summary

Treatment of non-small cell lung carcinoma (NSCLC) with immune checkpoint inhibitors (ICIs) has improved survival rates. However, ICIs can cause severe side effects leading to hospitalization and are typically managed with steroids. We reviewed how NSCLC patients with severe ICI toxicity are managed in real-world settings across UK hospitals and assessed the benefits they received from ICIs. Current treatment for ICI toxicity involves high-dose steroids, which can cause health complications. We aimed to refine this management approach by identifying specific side effects or patient groups that might benefit from lower steroid doses or early addition of other steroid-sparing agents. We also evaluated how stopping or continuing immunotherapy after recovering from side effects impacts lung cancer prognosis and the risk of future ICI toxicity. This is informative for clinicians as the decision to continue or stop ICI remains a very difficult clinical decision and an area that lacks clinical trial evidence.

## 1. Introduction

Immune checkpoint inhibitors (ICIs) have served as standard of care treatment for patients with recurrent or metastatic disease across multiple solid and hematologic malignancies. They involve interaction with immune checkpoint pathways such as the PD-1/PD-L1 axis, which plays a key role in tumor immune evasion. T cell activation is initiated when the T cell receptor (TCR) recognizes tumor-derived peptides presented by major histocompatibility complex (pMHC) molecules on the tumor surface [1]. However, PD-L1, expressed on tumor cells, binds to the PD-1 receptor on activated T cells, delivering an inhibitory signal that suppresses T cell activity and limits anti-tumor immunity [1,2]. Therapeutic blockade of this pathway using anti-PD-1 or anti-PD-L1 antibodies can contribute to restoration of T cell function and enhance immune-mediated tumor clearance [1]. In a distinct immunoregulatory mechanism, the CTLA-4 pathway regulates early T cell activation in lymphoid tissues. Effective T cell priming requires not only TCR engagement with pMHC, but also co-stimulatory signaling through CD28 binding to B7-1/2 ligands on antigen-presenting cells (APCs) [1]. CTLA-4, which is upregulated on activated T cells, competes with CD28 for B7-1/2 binding, thereby inhibiting T cell activation [1,3]. The use of anti-CTLA-4 antibodies blocks this inhibitory interaction, preventing CTLA-4 from outcompeting CD28, and thus facilitates T cell activation through positive co-stimulation [1].

ICIs such as anti-PD-L1 and anti-CTLA4 antibodies currently represent a widely adopted immunotherapy approach for patients with lung cancer [4]. The incorporation of these agents—either as monotherapy or in combination with chemotherapy—has led to improvements in most clinical efficacy endpoints compared to chemotherapy alone in patients with locally advanced or metastatic NSCLC lacking oncogenic driver alterations [5]. However, the increasing number of patients with non-small cell lung carcinoma (NSCLC) receiving ICIs has led to more patients developing immune-related adverse events (irAEs) in recent years. A meta-analysis of data from 38 trials of advanced-stage lung cancer patients reported incidences of Grade 3–5 irAEs for ICI monotherapy (6.6%), an ICI plus chemotherapy (11.4%), dual ICIs (13.8%), and dual ICIs plus chemotherapy (13.5%) [6]. Current ESMO Clinical Practice Guidelines suggest hospitalization for most patients experiencing Grade 3 or higher irAEs with corticosteroid treatment delivered as 1–2 mg/kg/day [7]. However, the impact of irAEs among NSCLC patients on treatment response to ICI therapy is currently limited. Additionally, there is a paucity of data regarding survival outcomes in the setting of immunotherapy following irAEs. These patients represent an important clinical population where real-world evidence regarding outcomes following the development of irAEs can help guide clinicians as to the optimal patient management strategy.

We conducted a multi-institutional review of NSCLC patients developing ICI-related toxicity and managed with corticosteroid agents. Our team aimed to evaluate treatment response, progression-free survival, and overall survival after onset of irAEs. We also assessed real-world use of corticosteroids and other novel immunosuppressive agents for the treatment of Grade 3 or higher ICI toxicities, where published data is currently lacking. The current management approach involves most patients being subjected to high-dose corticosteroids to treat irAEs, which itself is associated with significant side effects. We aimed to find groups of patients experiencing improved survival outcomes with specific doses of corticosteroid therapy to optimize therapeutic benefit. irAEs frequently necessitate cessation of ICIs, thereby restricting subsequent treatment options for NSCLC patients [8]. Therefore, we also hoped to clarify the clinical benefit of terminating vs. continuing ICI therapy following irAEs. Identification of such patients is important given the potential for long-term benefit and reduced side effects of ICIs compared to other systemic therapies. This would allow patients to continue ICIs to ensure greater duration of treatment benefit. We also aimed to identify patients who will not benefit from ICI continuation following irAEs, thereby aiding physicians to recommend other treatment options or terminating ICI therapy while transitioning to surveillance in cases of adequate disease control to avoid subsequent ICI-related toxicities.

## 2. Materials and Methods

### 2.1. Patient Selection

**Figure 1:**
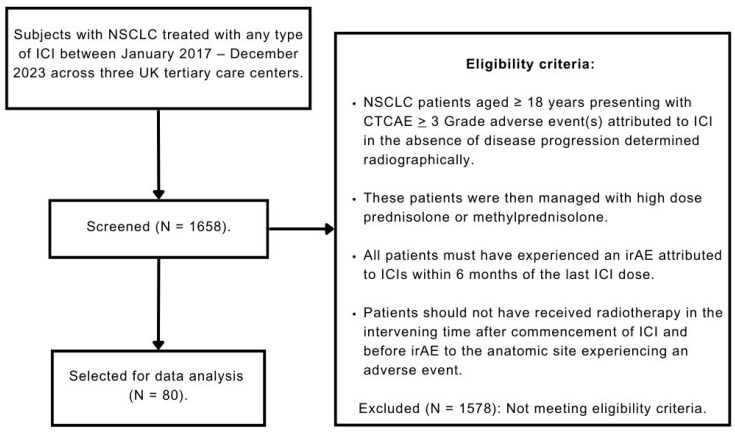
Flow diagram depicting the selection process of eligible patients for subsequent data analysis. Abbreviations: CTCAE, Common Terminology Criteria for Adverse Events; ICI, Immune Checkpoint Inhibitor; irAE, immune-related Adverse Event; NSCLC, Non-Small Cell Lung Carcinoma; UK, United Kingdom.

We screened adult NSCLC patients to identify eligible patients treated with high-dose corticosteroids for immunotherapy toxicity as described in Figure 1. We abstracted data from patient medical records, including clinicians’ notes, radiology, radiation oncology, operative, and pathology reports.

### 2.2. Statistical Considerations

The primary outcome measure was progression-free survival (PFS), defined as the interval from the onset of the 1st irAE to the earliest occurrence of radiological or clinical disease progression, last follow-up, or death. Overall survival (OS) was additionally assessed in the entire study cohort. Best overall response (BOR) to ICIs was determined based on individual treating physician assessments.

Median and interquartile ranges were used to summarize continuous variables of age, number of cycles, and duration of ICIs before 1st irAE, and duration of irAEs and weight-adjusted starting corticosteroid doses to treat irAEs. Frequency tables summarized categorical variables including gender, smoking status, stage at onset of 1st irAE, anatomic site of metastasis, mutational and programmed death-ligand 1 (PD-L1) status, ICI treatment line and type prior to 1st irAE, irAE grades and types, additional immunosuppressive agents used to treat irAEs, timeframe of 2nd irAE occurrence, and patterns of systemic treatment change after occurrence of irAEs.

Survival outcomes were assessed using the Kaplan–Meier method and Cox proportional hazards (CoxPH) models. Univariate and multivariate analyses were performed to evaluate associations with clinicopathological variables. Cox proportional hazards model assumptions were tested using Schoenfeld residuals. Only patterns of systemic treatment change after occurrence of irAEs demonstrated evidence of time-dependent effects, which were then modeled using a time-varying coefficient. The model included both the baseline and time-varying effects of patterns of systemic treatment change after occurrence of irAEs. Multivariate logistic regression was performed to assess the impact of baseline molecular variables on best overall response to ICI after 1st irAE. Treatment subgroups were compared using the Wilcoxon test. Statistical significance was defined as *p* < 0.05, with log-rank tests used to assess associations between patient, treatment, and irAE variables with both PFS and OS. Patients with missing data were excluded from analyses. All statistical analyses were conducted using R (version 4.2.3) in RStudio (version 2022.12.0.353) and Microsoft Excel (version 16.75).

## 3. Results

### 3.1. Patient Characteristics

We screened 1658 patients and identified 80 eligible individuals who received high-dose corticosteroids for immunotherapy-related severe adverse events. The median age at onset of 1st irAE was 69 years, and there was a relatively equal preponderance of male vs. female patients (Table 1). Most patients had a prior smoking history (*n* = 72, 91%), with the majority presenting with metastatic disease at onset of 1st irAE (*n* = 50, 63%), primarily with bone lesions (*n* = 20, 40%) (Table 1).

The majority of the patients underwent genomic profiling before 1st ICI therapy (*n* = 68, 85%), with most subjects harboring *KRAS* aberrations (*n* = 29, 36%) (Supplementary Table S1). PD-L1 Tumour Proportion Score (TPS) was also assessed in most patients (*n* = 77, 96%) (Supplementary Table S1).

Most patients developed their 1st irAE following treatment with their v ICI therapy (*n* = 71, 89%), mainly comprising of anti-PD-1 monotherapy (*n* = 40, 50%) (Supplementary Table S2). The median duration of the last ICI prior to onset of the 1st irAE was 2.76 months, and the patients received a median of four ICI cycles prior to onset of the 1st irAE (Supplementary Table S2).

An assessment of first irAEs demonstrated frequent onset of Grade 3 AEs (*n* = 74, 93%) mainly comprising colitis (*n* = 26, 33%), followed by pneumonitis (*n* = 14, 18%) and hepatitis (*n* = 13, 16%) (Supplementary Table S3). The median duration of 1st irAEs was 1.58 months (Supplementary Table S3). These were managed with a median starting corticosteroid dose of 60 mg/day, with a limited number of patients receiving additional immunosuppressive agents (*n* = 11, 14%) to manage their 1st irAEs (Supplementary Table S3). The patients received an additional immunosuppressive agent after a median number of 15 days had passed following the first dose of the corticosteroid regimen (Supplementary Table S3). Following the onset of the 1st irAEs, the most common pattern of treatment change involved permanent discontinuation of systemic therapy (*n* = 32, 40%) (Supplementary Table S3).

Patients developed up to two irAEs (*n* = 14, 18%) in the entire evaluated cohort. ICI rechallenge in 18 subjects led to 2nd irAEs in 7 (39%) patients. Most commonly occurring 2nd irAEs comprised Grade 3 AEs (*n* = 13, 93%), predominantly colitis (*n* = 5, 36%), followed by hepatitis (*n* = 4, 29%) and pneumonitis (*n* = 3, 21%) (Supplementary Table S4). The median duration of the 2nd irAEs was 0.76 months, which mainly occurred sequentially after the 1st irAE (*n* = 12, 86%) (Supplementary Table S4). Management typically involved initiation of corticosteroid therapy at a median dose of 72.5 mg/day, with none of the patients requiring further management with an additional immunosuppressive agent (Supplementary Table S4). Following the onset of the 2nd irAEs, the most common treatment modification was permanent discontinuation of systemic therapy (*n* = 8, 57%) (Supplementary Table S4).

### 3.2. Treatment Response and Survival Outcomes

**Figure 2:**
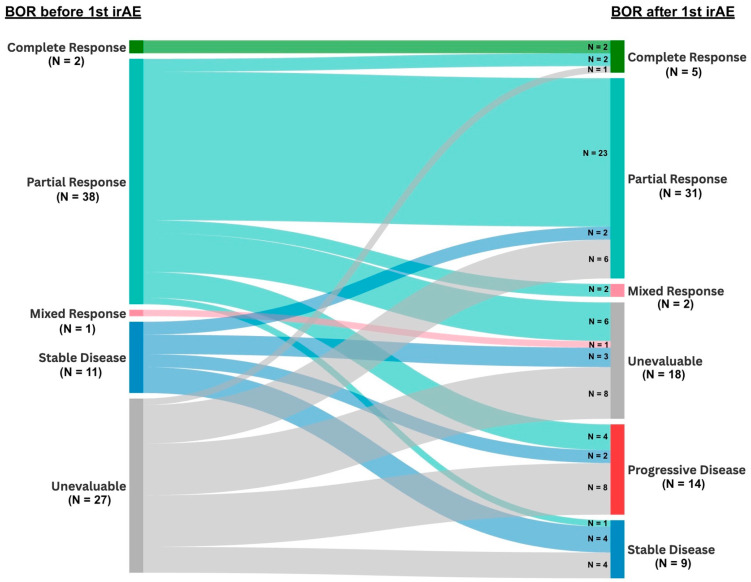
Sankey view comparing BOR to last immune checkpoint inhibitor therapy before and after onset of 1st irAE. Abbreviations: BOR, best overall response; irAE, immune-related Adverse Event.

We note that half of the cohort (*n* = 40, 50%) had achieved treatment response to ICIs prior to onset of the 1st irAE, with most patients (*n* = 25, 31%) retaining this response as their BOR after the 1st irAE (Figure 2). A limited number of patients with pre-1st irAE unevaluable response (*n* = 27, 34%) were successful in attaining response to ICI after onset of the 1st irAE (*n* = 7, 9%). No association was observed between the best overall response to ICI after the 1st irAE and *KRAS* mutation status (*p* = 0.89) or PD-L1 TPS (*p* > 0.05) (Supplementary Table S5).

For the complete cohort, the median PFS was 6.21 months (95% CI, 3.68–12.62), whereas the median OS was observed as 15.84 months (95% CI, 12.45–26.91). Analysis of patient-specific factors did not demonstrate any association of PFS with age at onset of 1st irAE (*p* = 0.20), gender (*p* = 0.40), disease stage (*p* = 0.37), *KRAS* mutational status (*p* = 0.12), PD-L1 TPS (*p* > 0.05) and baseline neutrophil-to-lymphocyte ratio (NLR) (*p* = 0.28) (Supplementary Figure S1A, Table 2). All of these patient-specific factors were not associated with OS, with the exception of low baseline NLR (*p* = 0.02) (Supplementary Figure S1B, Table 2).

**Figure 3:**
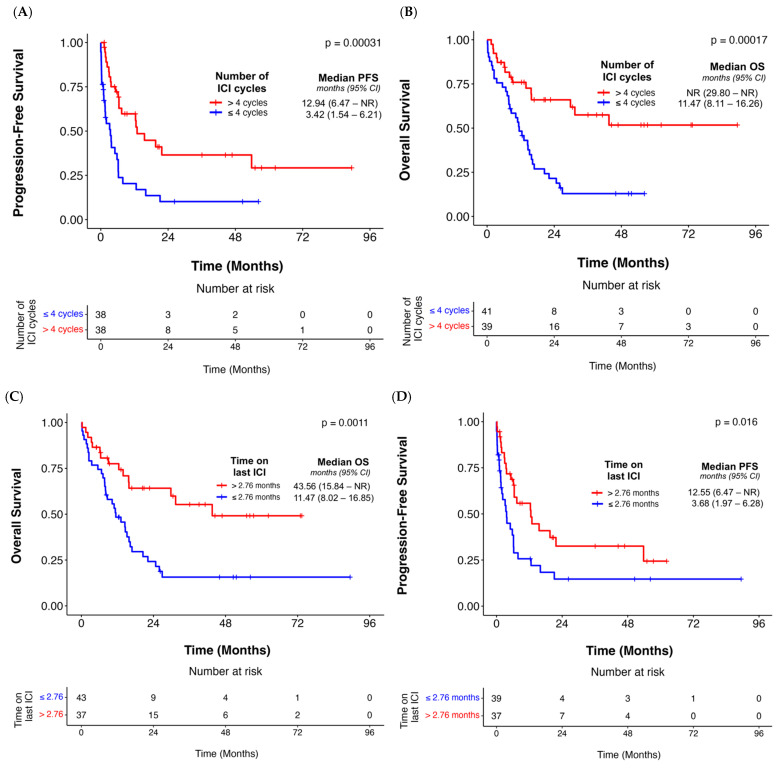
Associations between number of last ICI cycles before 1st irAE and PFS (**A**) and OS (**B**), respectively. The median value of 4 cycles was used to divide subjects in the entire evaluated cohort. Associations were also evaluated between the duration of the last ICI before 1st irAE and PFS (**C**) and OS (**D**), respectively. The median value of 2.76 months was used to divide subjects in this instance.

We also analyzed treatment-specific factors, where the patients receiving anti-PD-1 therapy with or without chemotherapy were not associated with PFS (*p* = 0.65) or OS (*p* = 0.43) (Table 2). However, the patients receiving >4 cycles of ICI prior to onset of the 1st irAE were associated with improved PFS (*p* < 0.001), as well as OS (*p* = 0.002) (Figure 3A,B, Table 2). We also observed an association between patients receiving ICI for a median duration exceeding 2.76 months prior to 1st irAE and PFS (*p* = 0.008) as well as OS (*p* = 0.004) (Figure 3C,D, Table 2).

Assessment of irAE-related factors with survival outcomes did not reveal an association of the total number and cumulative duration of all irAEs with PFS or OS (*p* > 0.05) (Table 3). Among the most frequently occurring 1st irAEs, ICI-induced pneumonitis was associated with the worst prognosis; PFS (*p* = 0.002) and OS (*p* < 0.001) (Supplementary Figure S2A,B, Table 3). Management of the 1st irAE with a median starting corticosteroid dose of ≤60 mg was not associated with PFS (*p* = 0.29), but it led to an improvement in OS (*p* = 0.04) (Supplementary Figure S3A,B, Table 3). However, the use of immunosuppressive agents in addition to corticosteroid regimens did not translate into an improvement in PFS (*p* = 0.93) or OS (*p* = 0.27) (Table 3). In the CoxPH model for PFS, the time-fixed effect of ICI resumption vs. discontinuation was not statistically significant (hazard ratio, 0.76 (95% confidence interval, 0.36–1.60; *p* = 0.47)), indicating no consistent effect over time. However, the time-varying component was significant (hazard ratio, 2.63 (95% confidence interval, 1.08–6.38; *p* = 0.03)), suggesting that the hazard of progression increased over time for patients who resumed ICI compared to those who discontinued systemic treatment. We did not note an association of OS among patients who resumed the same ICIs vs. those discontinuing systemic treatment altogether (*p* = 0.08) (Table 3). Lastly, we observed an association between BOR after onset of the 1st irAE and PFS (*p* < 0.001) as well as OS (*p* = 0.002) (Supplementary Figure S4A,B, Table 3). These were potentially driven in part by a lower baseline NLR (*p* = 0.046), a greater number of ICI cycles (*p* < 0.001), and duration of ICI regimen prior to onset of 1st irAE (*p* = 0.001) (Supplementary Figures S5–S7).

In the rechallenged subgroup of patients, seven (39%) patients developed a 2nd irAE comprising Grade 3 colitis (*n* = 4), Grade 3 pneumonitis (*n* = 2), and Grade 3 hepatitis (*n* = 1). Among these four colitis patients, two subjects had experienced Grade 3 colitis as their 1st irAE, and the remaining two had Grade 3 pneumonitis and hepatitis as their 1st irAE. Among the two pneumonitis patients, one subject had experienced Grade 3 pneumonitis as their 1st irAE, and the remaining patient had Grade 3 hepatitis as their 1st irAE. Finally, the Grade 3 hepatitis patient had also previously experienced this as their 1st Grade 3 irAE. Additionally, we did not observe an OS difference in rechallenged patients without a 2nd irAE vs. rechallenged subjects who did experience a 2nd irAE (median OS = not reached vs. 25.95 months, multivariate hazard ratio, 0.13 (95% CI, 0.01–1.64; *p* = 0.12).

## 4. Discussion

The research facet of immune checkpoint-induced toxicity represents a critical area of ongoing clinical research aimed at addressing a significant unmet need through the development of standardized treatment approaches that optimize survival outcomes. Development of irAEs can contribute to treatment interruption, prolonged and expensive emergency hospitalization, and reduced quality of life for NSCLC patients. Additionally, use of high-dose corticosteroids to treat irAEs itself can cause multiple side effects, with a current lack of consensus on the optimum starting corticosteroid dosing regimen. We endeavored to evaluate clinical outcomes following onset of ICI-related AEs in NSCLC patients who were then managed with corticosteroids with or without additional immunosuppressive agents, as data on this population is limited.

Meta-analyses of previous studies have demonstrated that NSCLC patients with a NLR of ≥5 (vs. <5) receiving PD-1 inhibitors (vs. PD-L1 inhibitors) or achieving response to ICIs (vs. non-responders) were at a higher risk of developing irAEs [9]. The vast majority of our patient population had received PD-1 inhibitors, with half of our cohort achieving ICI response prior to onset of the 1st irAE. In addition, we observed poor outcomes in patients with a high baseline NLR who developed irAEs. Our findings are also supported by previous studies suggesting that patients receiving their 1st ICI are more likely to experience a severe irAE leading to ICI discontinuation [10]. However, discontinuing ICIs following severe irAEs does not significantly impact treatment response and survival outcomes, albeit evaluated in an NSCLC population with a limited sample size [10]. Our data suggest that patients with a low baseline NLR receiving extended ICIs have a better prognosis. It also suggests careful management of severe irAEs with a starting corticosteroid dose of ≤60 mg/day when deemed clinically possible. Previous investigations have revealed that corticosteroid use can adversely impact OS and PFS of NSCLC patients, especially with anti-PD-1 therapy [11,12], which can be worse for patients with baseline steroid use prior to ICI initiation [13,14]. A previous study by Roboubi et al. demonstrated that poor PFS was associated with patients receiving systemic steroid doses ≥60 mg/day, albeit for the management of cancer-related symptoms [13]. However, in other cancer types such as melanoma, patients receiving prednisolone ≥60 mg/day for early onset irAE management experienced the worst post-irAE PFS and OS [15]. To our knowledge, our study is amongst the first to explore the role of starting corticosteroid dose for severe irAE management and patient survival outcomes in the context of NSCLC. Taken together, these findings emphasize the importance of judicious initial steroid dosing when managing severe irAEs to preserve long-term therapeutic benefits of ICIs.

Another novel strength of our study pertains to the observed association of >4 ICI cycles and >2.76 months of ICI treatment prior to the onset of irAEs with improved PFS and OS. This improvement in patient survival owing to prolonged ICI exposure may reflect a more sustained and effective immune-tumor interaction. A longer duration of immune checkpoint blockade before irAE development may enable a gradual and durable activation of T cell-mediated immunity, allowing for enhanced tumor surveillance and cytotoxicity prior to immune dysregulation [16,17]. Immune system “conditioning” over time through prolonged exposure to ICI may further promote maturation and expansion of tumor-reactive T cells, contributing to durable clinical benefit [18,19]. Additionally, the timing of irAE onset appears to be biologically meaningful; late-onset irAEs may serve as surrogate markers of therapeutic benefit, emerging after a period of immune priming and tumor-specific antigen recognition [20]. This may reflect an optimal immune-tumor balance, wherein anti-tumor immunity is effectively activated without early overactivation leading to severe autoimmunity. Moreover, patients who develop irAEs later in the treatment course may have reduced baseline immunosuppression (e.g., fewer regulatory T cells or myeloid-derived suppressor cells), thereby facilitating a more efficient and controlled immune response without triggering early toxicity [21,22,23,24]. Furthermore, the delayed use of corticosteroids in these patients likely reduces the risk of interrupting early immune priming, thus preserving ICI efficacy [25,26]. Finally, evolving changes in the tumor microenvironment, including increased infiltration of effector T cells, pro-inflammatory cytokines, and PD-L1 expression, may mediate both therapeutic response and subsequent immune toxicity, possibly in a time-dependent manner, supporting the notion that delayed irAE onset may reflect an evolving immune equilibrium favorable to survival [27,28].

We identified Checkpoint Induced Pneumonitis (CIP) as the poorest prognostic irAE, which is concordant with a previous study by Suresh et al. [29] highlighting the increased risk of mortality with onset of CIP, especially among adenocarcinoma patients. A possible physiological explanation of this observation is the consequential development of tissue hypoxia following onset of CIP. This can often contribute to failure of other organs due to low oxygenation, resulting in higher mortality than other irAEs. Additionally, similar to our findings, ICI alone or in combination with other agents was not associated with mortality. However, NSCLC patients on ICIs who do not develop irAEs have been historically observed to have poorer survival outcomes than those developing ≥Grade 2 irAEs [30]. Although CIP may have a better prognosis than individuals without any irAEs, careful surveillance of CIP patients is still warranted, especially in individuals with co-morbidities such as chronic obstructive pulmonary disease or interstitial lung disease.

In our study, while the average effect of ICI resumption was not significant, the significant time-varying interaction reveals that its impact on PFS is time-dependent. This suggests that initial benefits of resuming ICI may diminish over time, potentially due to acquired resistance or cumulative toxicity. In the context of the published literature, limited studies have specifically evaluated the efficacy of ICI rechallenge in patients with advanced-stage NSCLC. Mouri et al. conducted an analysis of 20 patients with advanced-stage NSCLC who underwent ICI rechallenge and found no significant difference in survival outcomes compared with 28 patients who discontinued ICIs [31]. Similarly, Santini et al. assessed NSCLC patients who initially responded to ICIs but later discontinued treatment due to irAEs, who demonstrated no clear survival benefit associated with rechallenge when compared with those who permanently ceased ICI therapy [32]. However, among patients who did not achieve a response prior to the onset of the irAE prompting treatment discontinuation, those who underwent rechallenge experienced improved PFS and OS relative to those who did not resume therapy [32]. In another small-cohort study of 52 patients who discontinued anti-PD-1 therapy owing to irAEs, 14 individuals were rechallenged with the same class of agents [33]. Although median PFS did not differ significantly between the rechallenge and non-rechallenge groups, median OS was significantly prolonged in the rechallenge cohort (not reached [95% CI, not estimable–not estimable] vs. not reached [95% CI, 8.4–not estimable]; *p* = 0.031) [33]. The overall conclusions from these studies remain equivocal, likely due to the retrospective nature of the analyses and the limited sample sizes. As such, identifying subgroups of patients who are most likely to benefit from ICI rechallenge should remain a priority in future prospective investigations.

This study is limited by heterogeneity in metastatic sites and variability in ICI treatment regimens, including differences in type, dosage, and schedule. Although our findings suggest that a lower initial dose of corticosteroid ≤60 mg was associated with improved survival, this observation may have been influenced by unmeasured confounding factors. In particular, patients requiring higher corticosteroid doses may have experienced more severe irAEs or had underlying autoimmune conditions that necessitated intensified immunosuppression. Additionally, early vs. late use of corticosteroids or longer duration of corticosteroid administration may have influenced the extent of immune suppression, thereby impacting survival independently of the initial corticosteroid dose. Additionally, the limited number of patients within certain subgroups may have resulted in suboptimal statistical power for some subtype-specific survival outcome analyses. Given the exploratory nature of this real-world evidence study, multiple testing adjustments were not performed. Therefore, caution is warranted while interpreting *p*-values from multivariable CoxPH analysis. Findings are considered hypothesis-generating and warrant validation in independent datasets. Our study also lacks biological data, as emerging evidence suggests the increasing role of utilizing blood-based products as potential biomarkers to identify high-risk candidates likely to develop ICI-induced toxicities. These have included the use of circulating immunophenotypes promoting high-grade and multiple irAEs [34,35], but the predictive role of circulating cytokines with respect to specific irAEs is currently a domain of active investigation.

## 5. Conclusions

Promising outcomes were seen in patients receiving longer treatment and achieving systemic ICI response, which contributes to favorable PFS and OS outcomes. While immunotherapy-related toxicity is a major concern, careful management strategies may allow select NSCLC patients to continue treatment and benefit from the long-term therapeutic effects of ICIs. Despite the inherent limitations of a retrospective design, these findings may inform clinical decision-making, particularly in selecting patients for ICIs who present with lower baseline levels of NLR. This would help limit immune-related toxicity and encourage their management with relatively lower corticosteroid doses to optimize clinical outcomes in this challenging patient population. Additionally, further clarification is required regarding the clinical benefit of terminating vs. continuing ICI therapy following irAEs. Future studies in larger, prospectively designed clinical trials are warranted to further validate these findings.

## Figures and Tables

**Table 1:** Baseline characteristics of the evaluated patients (*n* = 80).

	Total Number of Subjects	
	*n*	%
**Age at onset of 1st irAE**		
Median (years)	69	
Range (years)	38–81	
Interquartile range (years)	62–74	
**Gender**		
Male	41	51
Female	39	49
**Smoking Status**		
Non-smoker	7	9
Current Smoker	34	43
Former Smoker	38	48
Unknown	1	1
**Stage at onset of 1st irAE**		
Localized disease	30	38
Metastatic disease	50	63
**Anatomic sites of metastatic disease**		
Bone	20	40
Lymph node	13	26
Brain	10	20
Other ^a^	10	20
Liver	8	16
Adrenal gland	7	14
Pleural membrane	7	14

Abbreviation: irAE, immune-related adverse event. ^a^ spine (*n* = 2), left renal mass (*n* = 2), spleen (*n* = 1), abdominal deposits (*n* = 1), pectoralis minor muscle (*n* = 1), right psoas muscle (*n* = 1), right proximal humeral mass (n = 1), and gallbladder (*n* = 1).

**Table 2:** Associations of patient and treatment-specific factors with PFS and OS after onset of first irAE for the complete cohort.

	PFS				OS			
	Univariate HR (95% CI)	*p*-Value	Multivariate HR (95% CI) *	*p*-Value	Univariate HR (95% CI)	*p*-Value	Multivariate HR (95% CI) *	*p*-Value
**Age at onset of 1st irAE** (<69 years vs. ≥69 years)	0.69 (0.39–1.21)	0.20	N/A	N/A	0.57 (0.32–1.01)	0.06	N/A	N/A
**Gender** (Male vs. Female)	1.27 (0.73–2.22)	0.40	N/A	N/A	1.36 (0.77–2.38)	0.29	N/A	N/A
**Stage at time of onset of 1st irAE** (Localized vs. metastatic disease)	0.77 (0.43–1.36)	0.37	N/A	N/A	0.63 (0.34–1.14)	0.13	N/A	N/A
***KRAS*** (Mutant vs. wild type)	1.59 (0.81–3.14)	0.18	1.82 (0.86–3.86)	0.12	1.10 (0.57–2.09)	0.78	1.18 (0.57–2.43)	0.66
**PD-L1 TPS status**								
<1%	-	-	-	-	-	-	-	-
>1%–<50%	0.81 (0.34–1.93)	0.64	0.78 (0.31–1.92)	0.58	0.75 (0.34–1.65)	0.47	0.64 (0.27–1.49)	0.30
≥50%	0.84 (0.41–1.73)	0.63	0.86 (0.38–1.93)	0.71	0.61 (0.30–1.22)	0.16	0.80 (0.36–1.79)	0.59
**Baseline NLR** (≤6.50 vs. >6.50)	0.88 (0.46–1.69)	0.70	0.66 (0.31–1.40)	0.28	0.53 (0.28–0.99)	0.048	0.42 (0.20–0.87)	0.02
**ICI immediately preceding 1st irAE** (anti-PD-1 + chemotherapy vs. anti-PD-1 monotherapy)	0.97 (0.52–1.80)	0.91	0.85 (0.41–1.74)	0.65	1.50 (0.82–2.75)	0.19	1.31 (0.67–2.57)	0.43
**Number ICI cycles completed before 1st irAE** (>4 vs. ≤4 cycles)	0.37 (0.21–0.65)	<0.001	0.34 (0.18–0.62)	<0.001	0.32 (0.18–0.60)	<0.001	0.35 (0.18–0.67)	0.002
**Duration of last ICI treatment before 1st irAE** (>2.76 months vs. ≤2.76 months)	0.51 (0.29–0.89)	0.02	0.44 (0.24–0.81)	0.008	0.37 (0.20–0.69)	0.002	0.39 (0.20–0.74)	0.004

* adjusted analysis by age at onset of 1st irAE, gender, smoking status, and stage at time of onset of 1st irAE. Abbreviations: CI, confidence interval; HR, hazard ratio; ICI, immune checkpoint inhibitor; irAE, immune-related adverse event; *KRAS*, Kirsten rat sarcoma viral oncogene; N/A, not applicable; NLR, neutrophil to lymphocyte ratio; OS, overall survival; PD-L1, programmed death-ligand 1; PFS, progression-free survival; TPS, tumour proportion score.

**Table 3:** Associations of irAE-related factors with PFS and OS after onset of the 1st irAE for the complete cohort.

	PFS				OS			
	Univariate HR (95% CI)	*p*-Value	Multivariate HR (95% CI) *	*p*-Value	Univariate HR (95% CI)	*p*- Value	Multivariate HR (95% CI) *	*p*-Value
**Total number of irAEs** (>1 vs. 1)	0.97 (0.47–2.00)	0.94	1.06 (0.49–2.26)	0.89	0.93 (0.44–1.99)	0.86	1.05 (0.48–2.33)	0.90
**Cumulative duration of all irAEs** (>1.64 months vs. ≤1.64 months)	0.72 (0.40–1.29)	0.27	0.65 (0.35–1.22)	0.18	0.75 (0.42–1.36)	0.35	0.74 (0.39–1.41)	0.36
**Type of 1st irAE**								
Colitis vs. other irAEs	0.77 (0.41–1.46)	0.43	0.67 (0.34–1.33)	0.26	1.08 (0.59–1.96)	0.81	1.23 (0.64–2.34)	0.54
Hepatitis vs. other irAEs	1.06 (0.53–2.12)	0.87	1.29 (0.60–2.75)	0.51	0.74 (0.34–1.58)	0.43	0.96 (0.43–2.12)	0.92
Pneumonitis vs. other irAEs	2.92 (1.49–5.75)	0.002	3.21 (1.51–6.82)	0.002	3.39 (1.74–6.60)	<0.001	4.63 (2.17–9.90)	<0.001
**Starting corticosteroid dose** (≤60 mg vs. >60 mg)	0.67 (0.36–1.22)	0.19	0.71 (0.38–1.33)	0.29	0.56 (0.31–1.03)	0.06	0.52 (0.27–0.97)	0.04
**Additional immunosuppressive agents used to treat 1st irAE** (Yes vs. No)	0.94 (0.42–2.08)	0.87	0.96 (0.41–2.26)	0.93	0.53 (0.19–1.47)	0.22	0.54 (0.18–1.61)	0.27
**Patterns of treatment change after 1st irAE** (Resumed same ICI regimen vs. discontinued systemic therapy)	0.62 (0.30–1.29)	0.20	0.76 (0.36–1.60)	0.47	0.42 (0.19–0.96)	0.04	0.47 (0.21–1.09)	0.08
**Best overall response after 1st irAE** (Responders vs. Non-Responders)	0.23 (0.12–0.45)	<0.001	0.19 (0.09–0.40)	<0.001	0.38 (0.19–0.77)	0.007	0.32 (0.15–0.65)	0.002

* adjusted analysis by age at onset of 1st irAE, gender, smoking status, and stage at time of onset of 1st irAE. Abbreviations: CI, confidence interval; HR, hazard ratio; irAE, immune-related adverse event; OS, overall survival; PFS, progression-free.

## Data Availability

The original contributions presented in this study are included in the article/Supplementary Material; further inquiries can be directed to the corresponding authors.

## Supplementary Materials

The following supporting information can be downloaded at https://www.mdpi.com/article/10.3390/cancers17172819/s1, Supplementary Figure S1: Associations between baseline NLR prior to commencement of last ICI and PFS (A) and OS (B), respectively. The upper quartile value of 6.50 was used to divide subjects in the complete evaluated cohort. Supplementary Figure S2: Associations between pneumonitis vs other 1st irAEs and PFS (A) and OS (B), respectively. Supplementary Figure S3: Associations between starting corticosteroid dose to manage 1st irAE and PFS (A) and OS (B), respectively. The median value of 60 mg was used to divide subjects in each cohort. Supplementary Figure S4: Associations between best overall response on last ICI after 1st irAE and PFS (A) and OS (B), respectively. Patients achieving partial response were classified as responders whereas subjects attaining stable disease or progressive disease as their best overall response were categorized as non-responders. Supplementary Figure S5: Boxplot of patients’ baseline NLR prior to commencing last ICI with respect to the best overall response to last ICI after 1st irAE. Supplementary Figure S6: Boxplot of patients’ number of ICI cycles before 1st irAE with respect to the best overall response to last ICI after 1st irAE. Supplementary Figure S7: Boxplot of patients’ duration of last ICI prior to onset of 1st irAE with respect to the best overall response to last ICI after 1st irAE. Supplementary Table S1: Mutational profile and PD-L1 TPS prior to commencing 1st line ICI, among evaluated patients (N = 80). Supplementary Table S2: Immune checkpoint inhibitor treatment details among evaluated subjects (N = 80). Supplementary Table S3: Overview of 1st irAEs among the evaluated cohort (N = 80). Supplementary Table S4: Overview of 2nd irAEs among the evaluated cohort (N = 14). Supplementary Table S5: Multivariate logistic regression analysis of NSCLC subjects with respect to ICI response adjusted by age at onset of 1st irAE, gender, smoking status and stage at time of onset of 1st irAE.
