# Supplementary material for: Predictors and Outcomes of Non-Small Cell Lung Carcinoma Patients Following Severe Immune Checkpoint Inhibitor Toxicity: A Real-World UK Multi-Centre Study
Source: Cancers (Basel). 2025 Aug 28;17(17):2819. doi: 10.3390/cancers17172819 (PMC12427550; doi:10.3390/cancers17172819)
Supplement: Supplementary file 1 [file cancers-17-02819-s001.zip › Supplementary Figures.pdf]

A

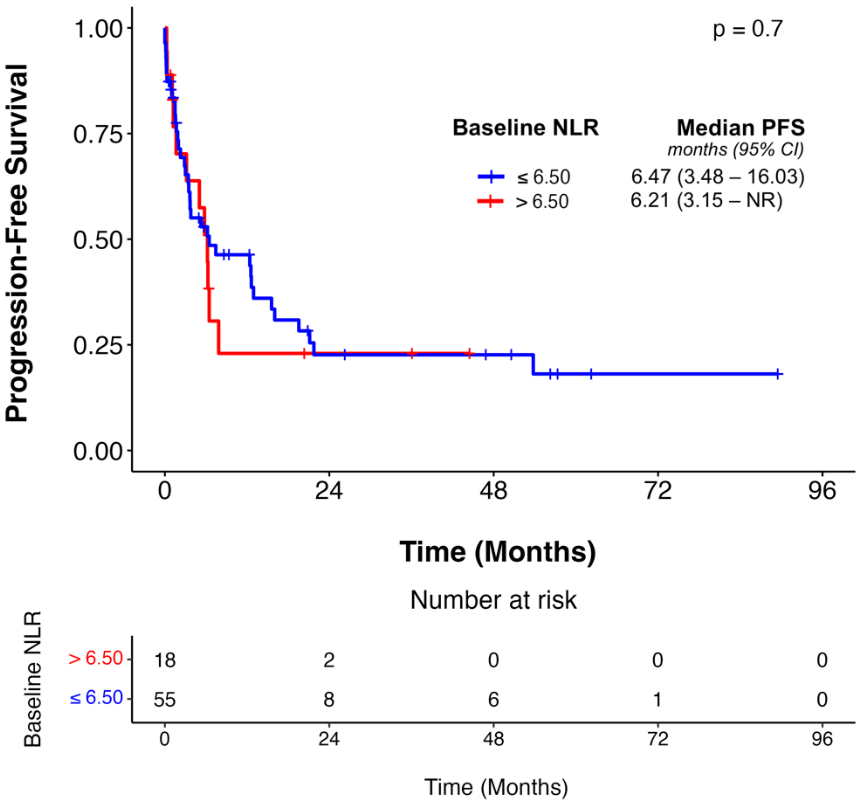

B

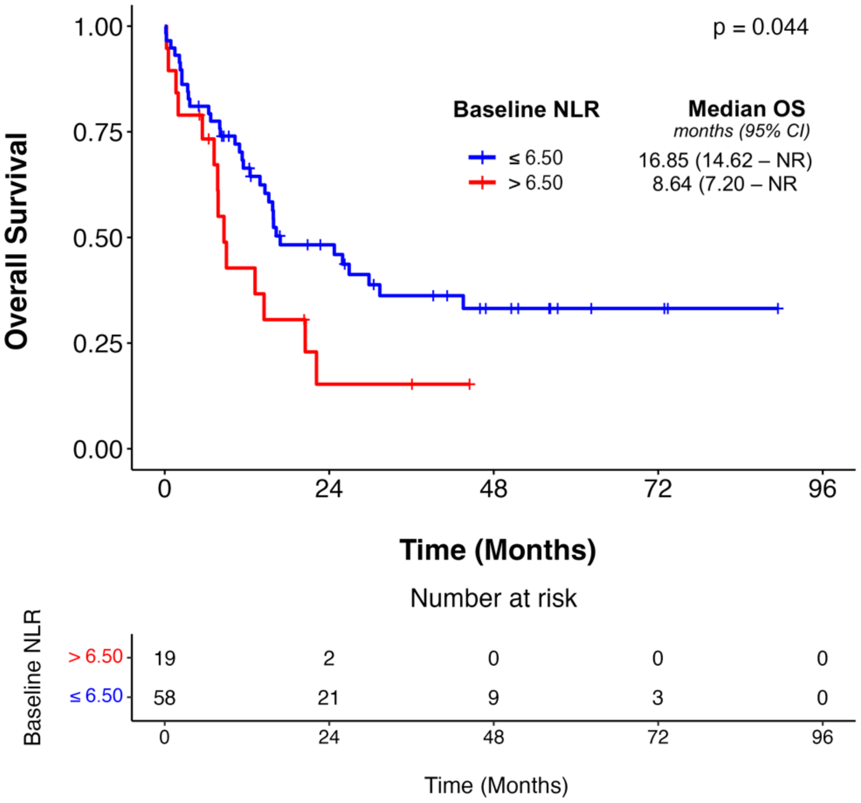

Supplementary Fig. S1: Associations between baseline NLR prior to commencement of last ICI and PFS (A) and OS (B), respectively. The upper quartile value of 6.50 was used to divide subjects in the complete evaluated cohort.

A

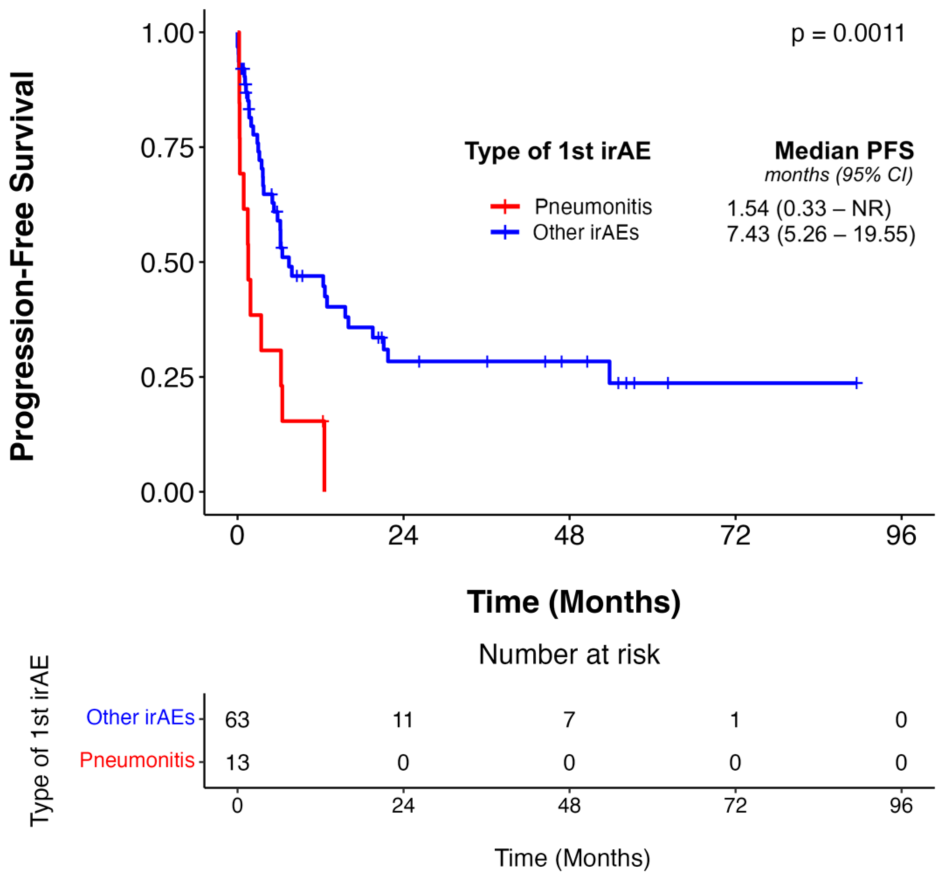

B

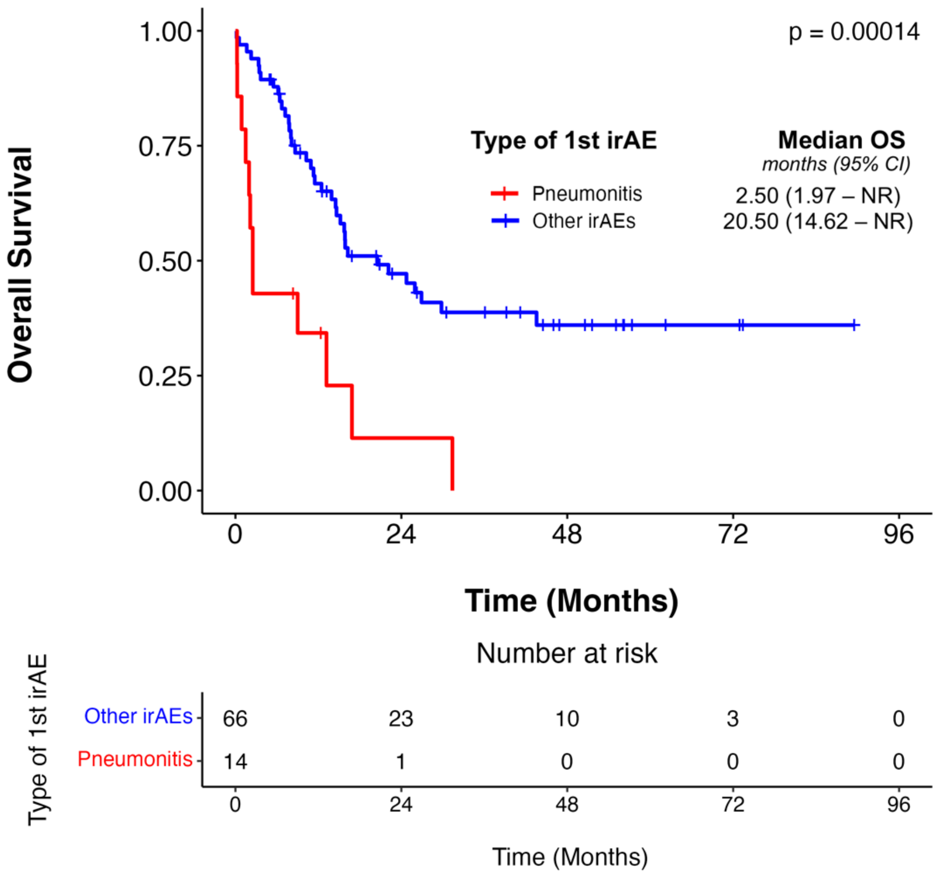

Supplementary Fig. S2: Associations between pneumonitis vs other 1st irAEs and PFS (A) and OS (B), respectively.

A

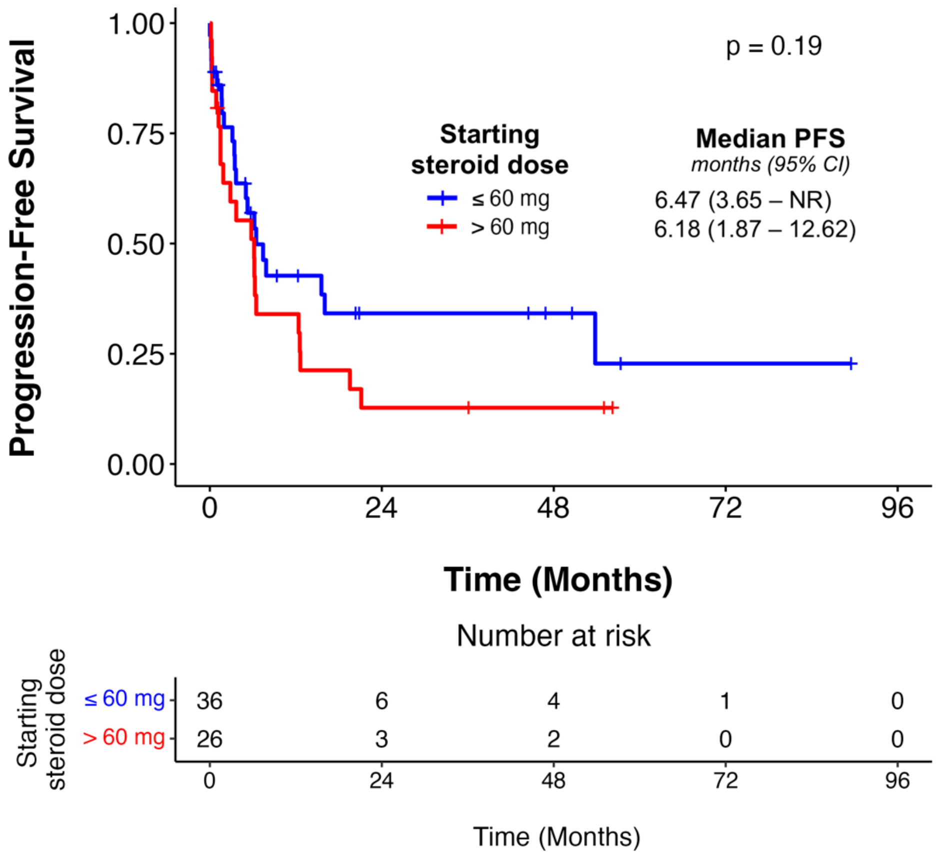

B

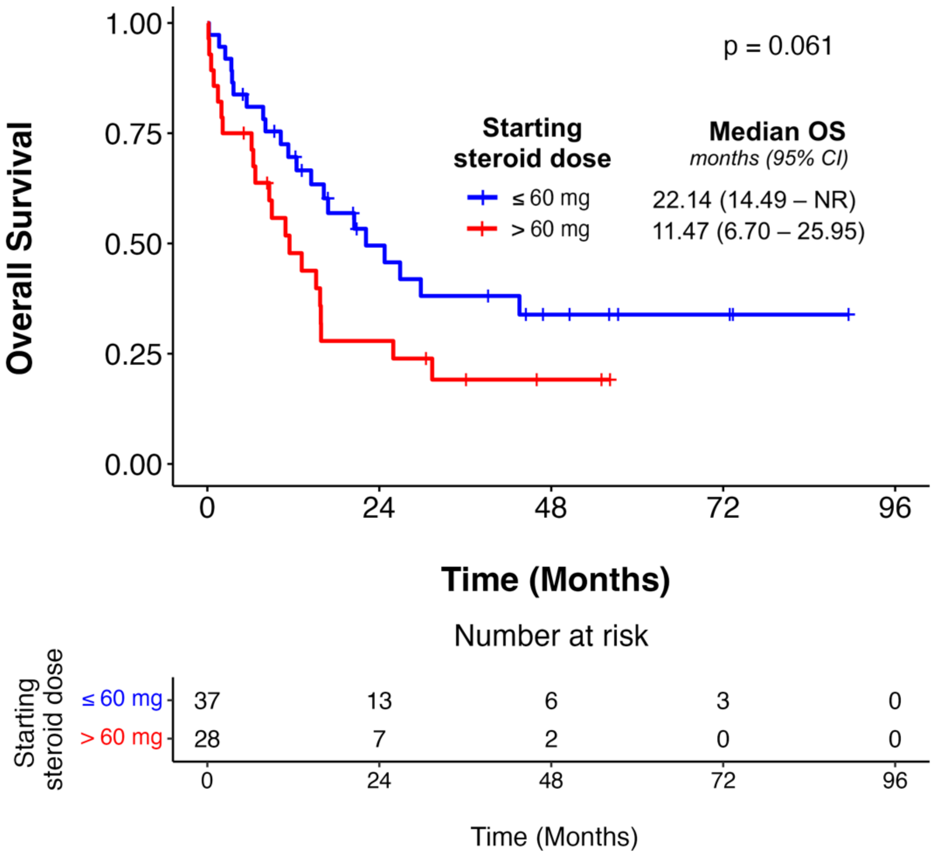

Supplementary Fig. S3: Associations between starting corticosteroid **dose to manage 1st irAE and PFS (A) and OS (B), respectively. The median value of 60 mg was used to divide subjects in each cohort.**

A

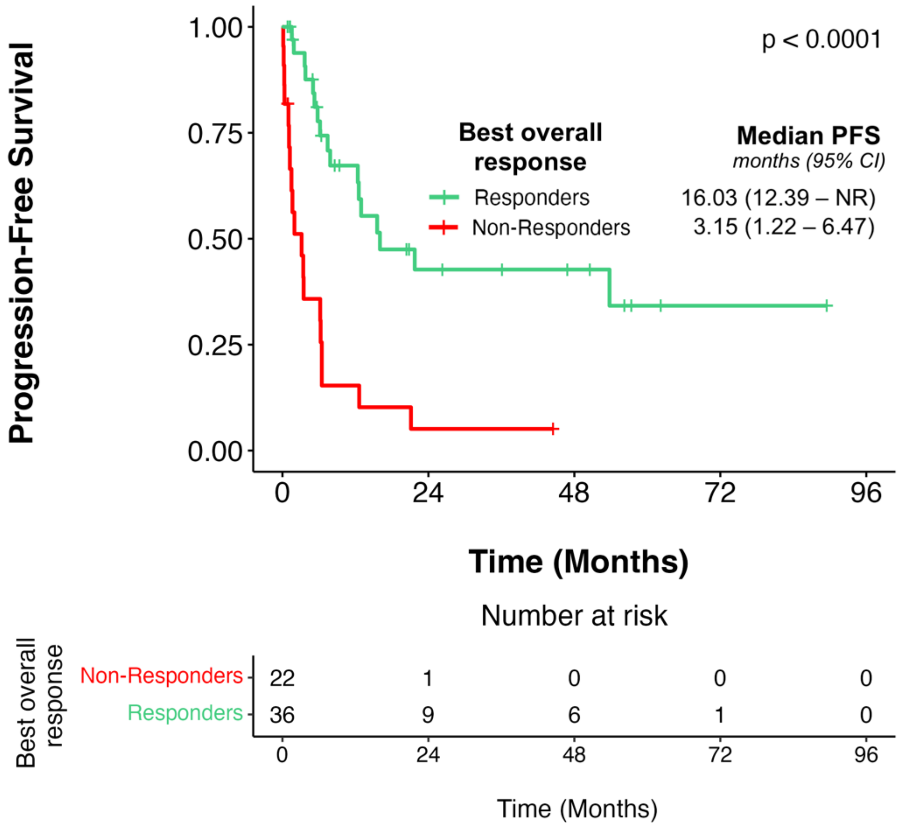

B

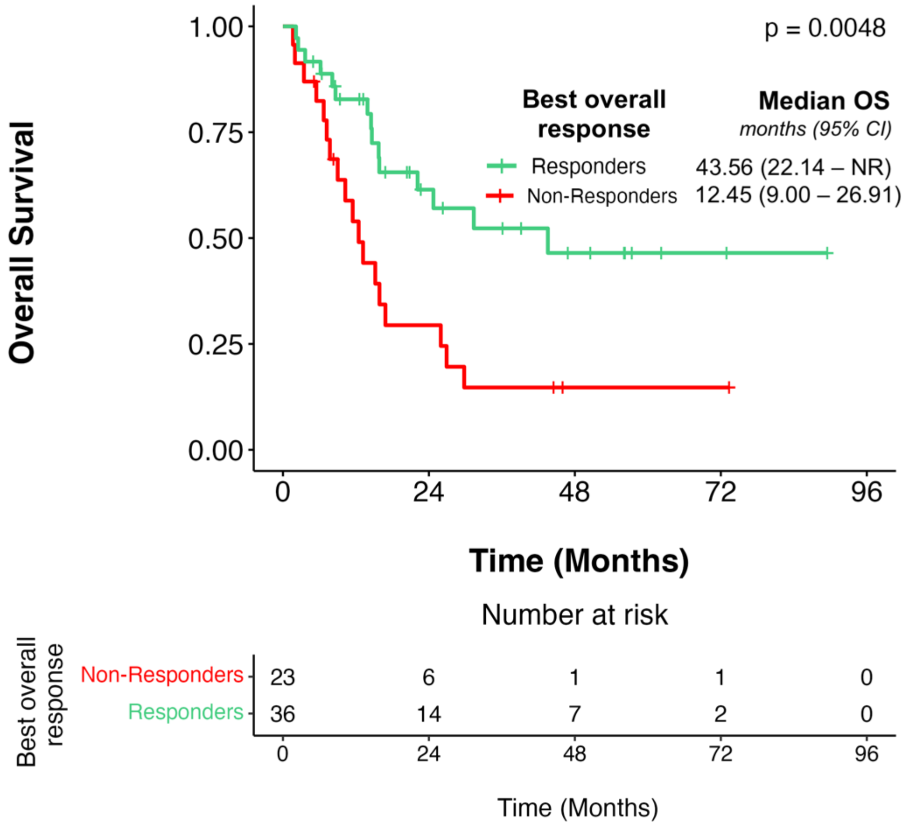

Supplementary Fig. S4: Associations between best overall response on last ICI after 1st irAE and PFS (A) and OS (B), respectively. Patients achieving partial response were classified as responders whereas subjects attaining stable disease or progressive disease as their best overall response were categorized as non-responders.

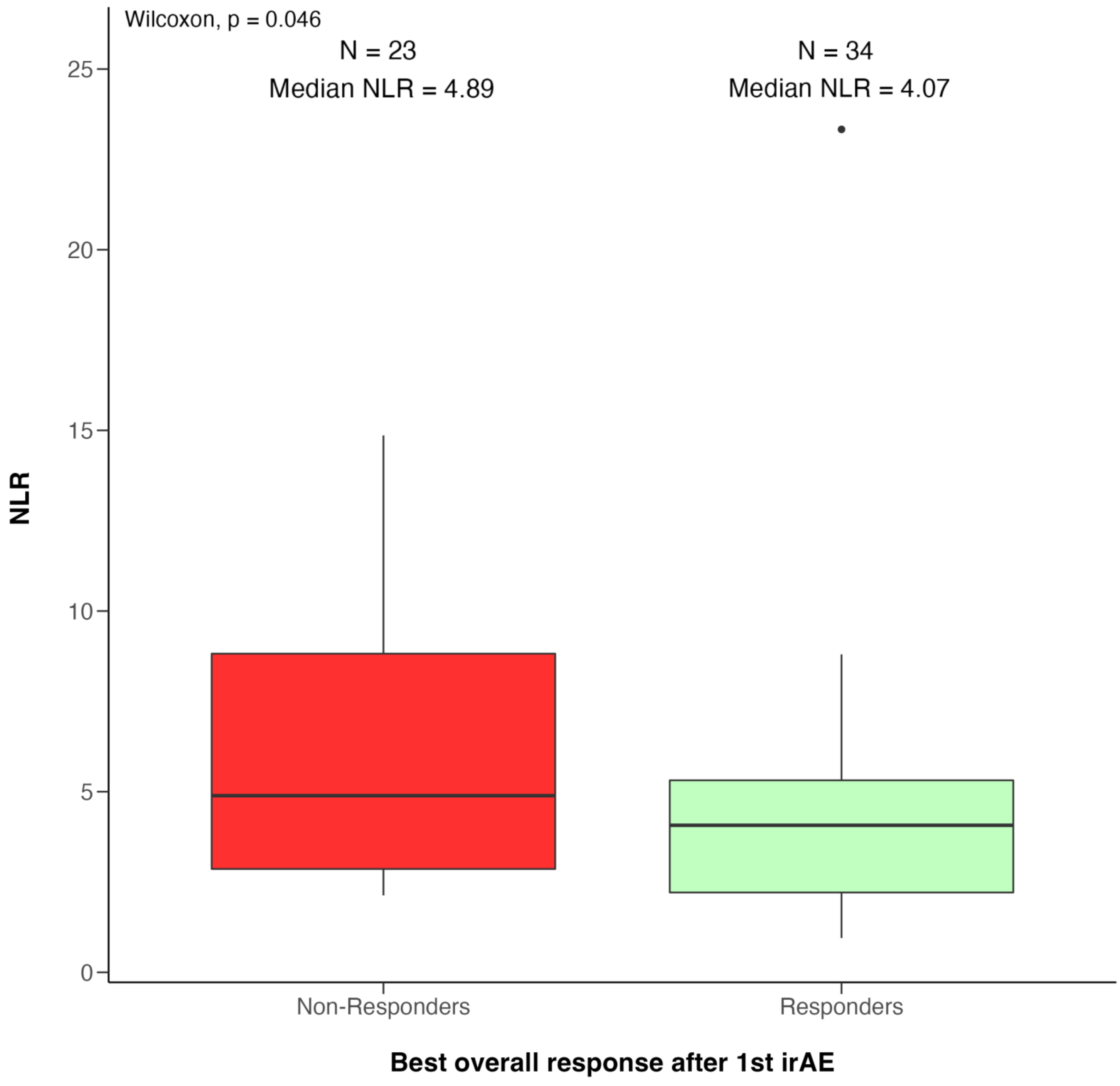

Supplementary Figure S5: Boxplot of patients' baseline NLR prior to commencing last ICI with respect to the best overall response to last ICI after 1st irAE.

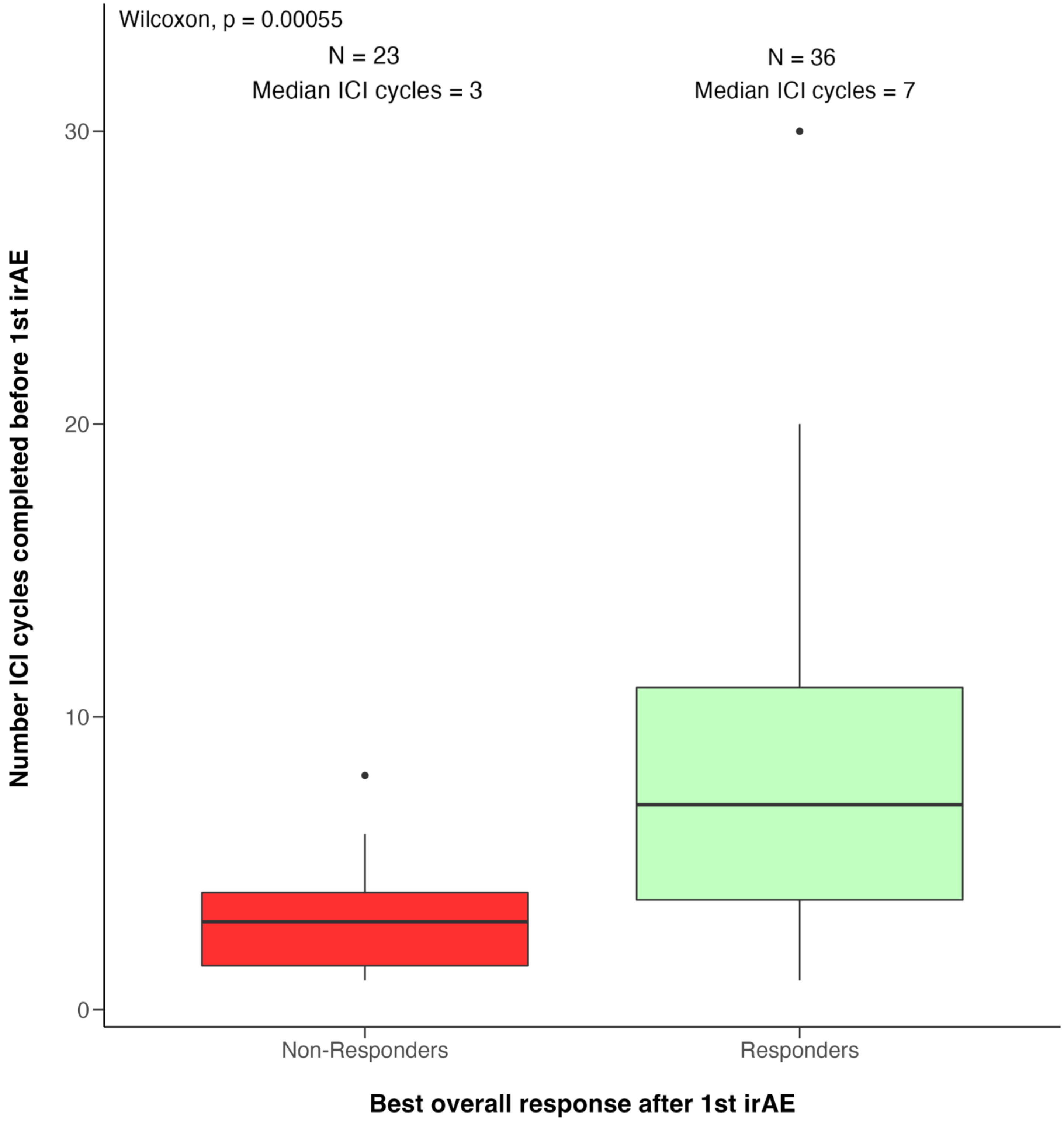

Supplementary Figure S6: Boxplot of patients' number of ICI cycles before 1st irAE with respect to the best overall response to last ICI after 1st irAE.

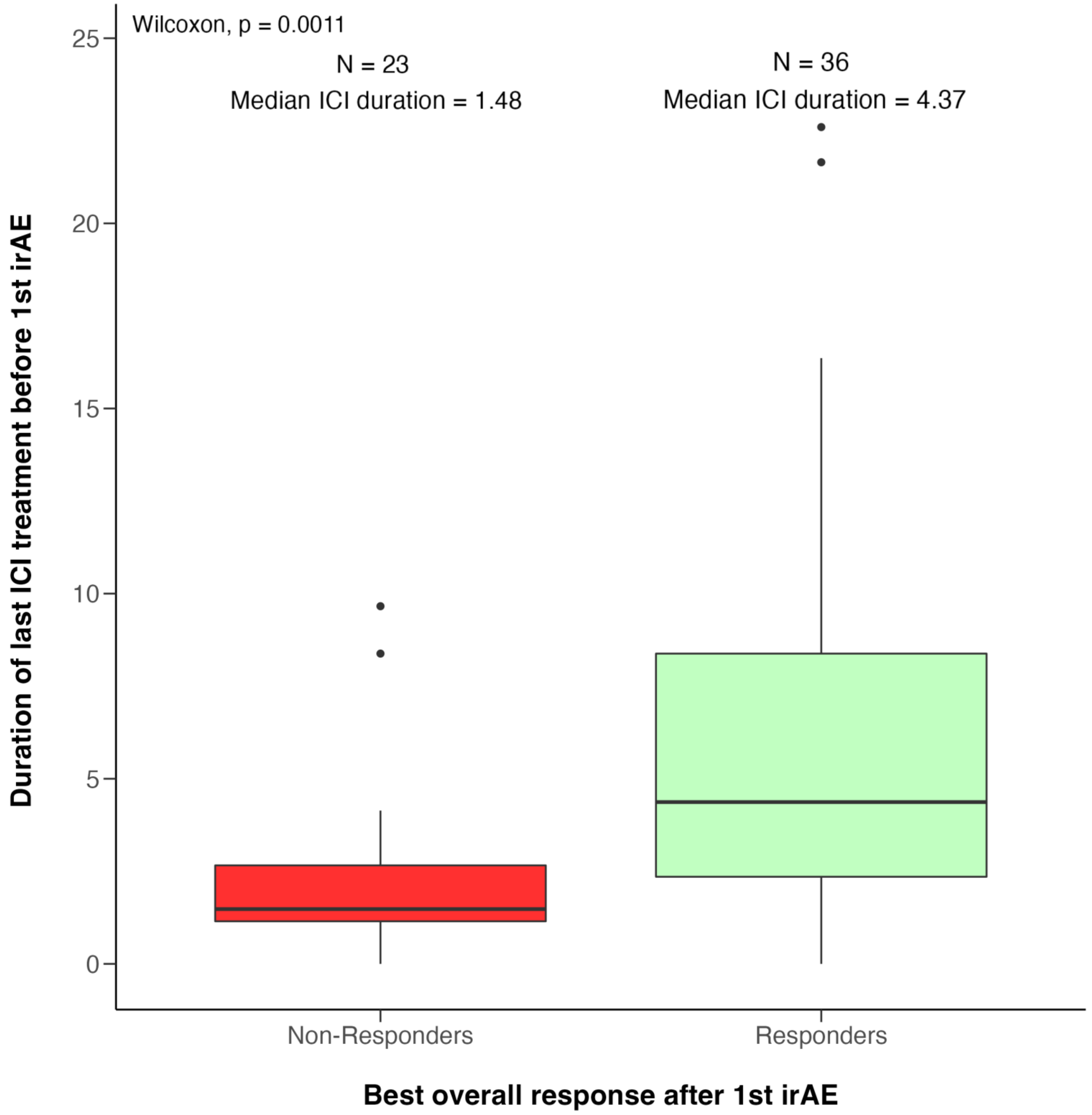

Supplementary Figure S7: Boxplot of patients' duration of last ICI prior to onset of 1st irAE with respect to the best overall response to last ICI after 1st irAE.
