# Supplementary material for: Predictors and Outcomes of Non-Small Cell Lung Carcinoma Patients Following Severe Immune Checkpoint Inhibitor Toxicity: A Real-World UK Multi-Centre Study
Source: Cancers (Basel). 2025 Aug 28;17(17):2819. doi: 10.3390/cancers17172819 (PMC12427550; doi:10.3390/cancers17172819)
Supplement: Supplementary file 1 [file cancers-17-02819-s001.zip › Supplementary Tables.pdf]

Supplementary Tables

Supplementary Table S1: Mutational profile and PD-L1 TPS prior to commencing 1st line ICI, among evaluated patients (N = 80).

	**Total number of subjects**	
	**N**	**%**
**Mutation status**		
KRAS +	29	36
KRAS -	30	38
Unknown KRAS status	21	26
EGFR +	4	5
EGFR -	62	78
Unknown EGFR status	14	18
ALK +	0	0
ALK -	67	84
Unknown ALK status	13	16
BRAF +	2	3
BRAF -	48	60
Unknown BRAF status	30	38
**PD-L1 TPS status**		
Available	77	96
Unknown	3	4

Abbreviations: ALK, anaplastic lymphoma kinase; BRAF, v-raf murine sarcoma viral oncogene homolog B1; EGFR, epidermal growth factor receptor; KRAS, kirsten rat sarcoma viral oncogene; PD-L1, programmed death-ligand 1; TPS, tumour proportion score.

Supplementary Table S2: Immune checkpoint inhibitor treatment details among evaluated subjects (N = 80).

	Total number of subjects	
	**N**	**%**
**ICI treatment line setting preceding 1st irAE**		
1st line ICI	71	89
2nd line ICI	8	10
Adjuvant ICI	1	1
**ICI immediately preceding 1st irAE**		
Anti-PD-1 monotherapy	40	50
Anti-PD-1 and chemotherapy	32	40
Anti-PD-1 and anti-CTLA-4 therapy	1	1
Anti-PD-L1 monotherapy	4	5
Anti-PD-L1, chemotherapy and targeted therapy	3	4
**Duration of last ICI treatment before 1st irAE**		
Median (months)	2.76	
Interquartile range (months)	1.26 – 5.77	
**Number of ICI cycles completed before 1st irAE**		
Median	4	
Range	1 – 30	

Abbreviations: CTLA-4, cytotoxic T lymphocyte antigen-4; ICI, immune checkpoint inhibitor; irAE, immune-related adverse event; PD-1, programmed cell death-1; PD-L1, programmed death-ligand 1.

Supplementary Table S3: Overview of 1st irAEs among the evaluated cohort (N = 80).

	Total number of subjects	
	N	%
**Grade of 1st irAE**		
Grade 3	74	93
Grade 4	5	6
Grade 5	1	1
**Type of 1st irAE**		
Colitis	26	33
Pneumonitis	14	18
Hepatitis	13	16
Acute kidney injury	7	9
Rash	5	6
Gastritis	2	3
Arthritis	2	3
Bullous pemphigoid	1	1
Cardiotoxicity (bradycardia)	1	1
Cholangitis	1	1
Diarrhea (secondary to enteritis)	1	1
Mucositis	1	1
Myocarditis	1	1
Myositis	1	1
Pancreatitis	1	1
Proctitis	1	1
Retinopathy	1	1
Vomiting secondary to sclerosing cholangitis	1	1
**Duration of 1st irAE**		

Median (months)	1.58	
Interquartile range (months)	0.61 – 2.88	
**Starting corticosteroid dose to treat 1st irAE (mg/day)**		
Median	60	
Range	30 – 1000	
**Additional immunosuppressive agents used to treat 1st irAE**		
None	69	86
Infliximab	6	8
Infliximab and vedolizumab	1	1
Cyclophosphamide	1	1
Methotrexate	1	1
Clobetasol propionate	1	1
Tacrolimus	1	1
**Time of prescribing additional immunosuppressive agent after commencing corticosteroid regimen**		
Median (days)	15	
Interquartile range (days)	0 - 108	
**Patterns of treatment change after 1st irAE**		
Resumed same ICI regimen	19	24
Switched to chemotherapy	8	10
Switched to chemotherapy and targeted agents	1	1
Discontinued systemic therapy	32	40
Received RT only to treat limited remaining burden of disease	1	1
Received RT only due to PD	6	8
Received chemotherapy only due to PD	8	10
Received targeted agents only due to PD	2	3
Switched to chemotherapy and targeted agents due to PD	1	1
Received RT and surgery due to PD	1	1
Received RT and chemotherapy due to PD	1	1
Resumed treatment (chemotherapy only) after onset of 2nd irAE	1	1

Abbreviations: ICI, immune checkpoint inhibitor; irAE, immune-relate adverse event; RT, radiotherapy; PD, progressive disease.

Supplementary Table S4: Overview of 2nd irAEs among the evaluated cohort (N = 14).

	Total number of subjects	
	**N**	**%**
**Grade of 2nd irAE**		
Grade 3	13	93
Grade 4	1	7
**Type of 2nd irAE**		
Colitis	5	36
Hepatitis	4	29
Pneumonitis	3	21
Acute kidney injury	1	7
Pericarditis	1	7
**Duration of 2nd irAE**		
Median (months)	0.76	
Interquartile range (months)	0.35 – 2.19	
**Timeframe of 2nd irAE occurrence**		
Sequentially after 1st irAE	12	86
Concurrently with 1st irAE	2	14
**Starting corticosteroid dose to treat 2nd irAE (mg/day)**		
Median	72.5	
Range	55 – 364	
**Additional immunosuppressive agents used to treat 2nd irAE**		
None	14	100
**Patterns of treatment change after 2nd irAE**		
Resumed same ICI regimen	1	7
Switched to chemotherapy	1	7

Switched to targeted agents	1	7
Discontinued systemic therapy	8	57
Received RT only due to PD	2	14
Received chemotherapy only due to PD	1	7

Abbreviations: ICI, immune checkpoint inhibitor; irAE, immune-relate adverse event; RT, radiotherapy; PD, progressive disease.
